# The Role of TcdB and TccC Subunits in Secretion of the *Photorhabdus* Tcd Toxin Complex

**DOI:** 10.1371/journal.ppat.1003644

**Published:** 2013-10-03

**Authors:** Guowei Yang, Nicholas R. Waterfield

**Affiliations:** 1 MOH Key Laboratory of Systems Biology of Pathogens, Institute of Pathogen Biology, Chinese Academy of Medical Sciences & Peking Union Medical College, Beijing, People's Republic of China; 2 The Division of Microbiology and Infection, Warwick Medical School, Warwick University, Coventry, United Kingdom; Massachusetts General Hospital, Harvard Medical School, United States of America

## Abstract

The Toxin Complex (TC) is a large multi-subunit toxin encoded by a range of bacterial pathogens. The best-characterized examples are from the insect pathogens *Photorhabdus*, *Xenorhabdus* and *Yersinia*. They consist of three large protein subunits, designated A, B and C that assemble in a 5∶1∶1 stoichiometry. Oral toxicity to a range of insects means that some have the potential to be developed as pest control technology. The three subunit proteins do not encode any recognisable export sequences and as such little progress has been made in understanding their secretion. We have developed heterologous TC production and secretion models in *E. coli* and used them to ascribe functions to different domains of the crucial B+C sub-complex. We have determined that the B and C subunits use a secretion mechanism that is either encoded by the proteins themselves or employ an as yet undefined system common to laboratory strains of *E. coli*. We demonstrate that both the N-terminal domains of the B and C subunits are required for secretion of the whole complex. We propose a model whereby the N-terminus of the C-subunit toxin exports the B+C sub-complex across the inner membrane while that of the B-subunit allows passage across the outer membrane. We also demonstrate that even in the absence of the B-subunit, that the C-subunit can also facilitate secretion of the larger A-subunit. The recognition of this novel export system is likely to be of importance to future protein secretion studies. Finally, the identification of homologues of B and C subunits in diverse bacterial pathogens, including *Burkholderia* and *Pseudomonas*, suggests that these toxins are likely to be important in a range of different hosts, including man.

## Author Summary

The Toxin Complex (TC) is a large multimeric protein complex first identified in the insect pathogens *Photorhabdus* and *Xenorhabdus*. TC isolates from these pathogens exhibit oral toxicity to a diverse range of insects. As such there is significant interest in developing them as candidates for crop protection strategies. Currently all insect resistant transgenic crops rely upon the production of *Bacillus thuringiensis* Cry toxins. However, to minimise the risk of insect resistance development it is imperative to develop additional toxin systems employing alternative modes of action. A barrier to the further development of TCs as agrochemical tools has been the complexity of their synthesis, secretion and assembly. Little is known about how the large TC subunits are secreted across the bacterial cell wall. We present here an investigation into the roles that the different domains of the B and C-subunit proteins play in secretion of the whole TC. The significance of this goes beyond these specific insect toxins as homologues of these two subunits are encoded in the genomes of a range of human pathogens, such as *Burkholderia* and *Yersinia*, in which they have been implicated in human virulence.

## Introduction

*Photorhabdus* are Enterobacteriaceae which live in an obligate mutualistic association with entomopathogenic nematodes (*Heterorhabditis*), which invade and kill insects in the soil [1]. Upon invasion of the insect's open blood system the nematode regurgitates *Photorhabdus* bacteria which release a plethora of toxins to kill the insect and protect the cadaver from invading scavengers and saprophytes [1]. An important class of secreted toxins are the Toxin Complexes (TC) [2], [3], [4]. These TC toxins constitute large multimeric protein complexes, some of which have been shown to exhibit oral toxicity to a range of insects [5], [6]. This has made them potential candidates to augment the successful *Bacillus thuringiensis* Crystal-toxin crop protection technology. TCs were first characterized in the insect pathogens *Photorhabdus* and *Xenorhabdus* although it has now become clear that *tc* gene homologues are in fact widely distributed in a range of other pathogens [2]. These include the Gram-negative human disease agents such as *Yersinia* and *Burkholderia* and Gram-positive insect pathogens such as *Paenibacillus* and *B. thuringiensis* strain IBL200 (accession NZ_ACNK01000119). While the TCs of *Yersinia entomophaga* are active against insects [7], homologues in other members of the genus, such as *Yersinia pseudotuberculosis*, appear to be adapted to act upon the mammalian gut [8]. They have also been implicated in mammalian gut colonisation in at least one strain of *Y. enterocolitica* [9].

The TC is a large multi-subunit toxin comprising three subunits, exemplified by the *P. luminescens* proteins TcdA, TcdB and TccC (from here on also referred to as A, B and C-subunits [10]). The subunits themselves are large proteins with the examples of TcdA1, TcdB1 and TccC5 being 2517 aa (283 kDa), 1477 aa (165 kDa) and 939 aa (105 kDa) respectively. In the *Xenorhabdus nematophila* TC, which consists of, XptA2, XptB1 and XptC1, the subunits apparently assemble in a 4∶1∶1 stoichiometry respectively. In this complex the A-subunits appear to form a tetramer of around 1120 kDa that is able to associate with a tightly bound 1∶1 sub-complex of the B and C-subunits [11], [12]. Interestingly in *Y. entomophaga*, the TC structure is predicted to show five-fold symmetry and to also associate with a chitinase enzyme [7]. More recently a high resolution cryo-EM structural model has been proposed to describe the structure and conformational changes that the TcdA1 subunit pentomer can undergo. It is shown to likely perform an “injection-like” process which presumably facilitates the delivery of the toxic B+C sub-complex into the host cell [13].

There has been some progress ascribing biological function to certain TC subunits and domains. For example it has been shown that the *Xenorhabdus* A-subunits encode a host gut cell receptor binding function, targeted to the membranes of insect brush border cells. Two different A-subunits were shown to ascribe different species specificities. [12], [14]. Furthermore, various sub-domains of *Photorhabdus* TC proteins have been investigated by transient expression in transfected mammalian cells [3]. More recently Lang *et al* demonstrate the mode of action of certain C-subunit C-terminal domains, which cause ADP-ribosylation of actin and RhoA [14]. The C-subunit family proteins all have a common conserved N-terminus and highly variable C-terminal domains. This bi-partite structure is now recognized as a common theme in “polymorphic toxin systems”. That is, many toxin families are seen to contain conserved N-terminal domains, which interact with various secretion systems, yet possess interchangeable and highly variable C-terminal “toxic” domains [15], [16], [17]. Certain regions of TC subunit proteins do exhibit homology to certain non-TC proteins. These include the first 361 amino acids (aa) of the TcdB1 B-subunit, which shows good homology to the N-terminus of the secreted *Salmonella* toxin, SpvB [18]. In addition aa154–290 of the TcdA1 A-subunit shows homology to SpvA protein. Like the A and B subunit genes, the *spvA* and *spvB* genes are also tightly linked, in this case on the *Salmonella* virulence plasmid [19]. Furthermore, the C-subunit proteins belong to a much larger family which includes the enigmatic Rhs proteins first discovered in *E. coli* [20].

Homologues of B and C-subunit genes are frequently seen tightly linked in other bacteria, often in the absence of an A-subunit homologue. Furthermore in strains of *Burkholderia* and *Pseudomonas*, homologues of B and C genes are present as genetic fusions, comprising a single long open reading frame. Indeed the increasing numbers of genome sequencing projects have revealed more distant *tc* B+C homologues in bacteria as diverse as *Wolbachia*, *Mycobacteria*, *Plesiocystis pacifica* and even fungi including *Gibberella zeae* and *Podospora anserine*. This supports a much wider role for these protein families beyond insect toxicity. Conversely, A-subunit gene homologues (exemplified by *TcdA1* and *TcaAB* like proteins) are typically only seen encoded in genomes that also have B and C gene homologues. This suggests that the B and C sub-complex plays a central role in the biological activity of the TC while the A-subunits facilitate more host specific roles. Evidence suggests that the A-subunit is most likely a combined host-cell targeting and B+C subunit delivery system [13] [21]. It should be noted that when heterologously expressed at high levels, the A-subunit and B+C sub-complex have been shown to exhibit limited oral toxicity independently of one another, although together they form a far more potent complex [3], [21], [22]. The production of the TcdA1 protein in transgenic plants [23] also reconstituted partial activity, suggesting these proteins could provide a potential alternative or addition to the well-established *cry*-toxin pest control technology.

Heterologous expression studies of TC homologues from species other than *Photorhabdus* and *Xenorhabdus* have also been published, including those of *Serratia entomophila*, *Y. pseudotuberculosis* and *Y. pestis* [8], [24], [25]. Furthermore, the heterologous production of TC toxins in *Enterobacteria* species which associate with termites has also been explored as a novel biological control strategy [26].

Recent work in our laboratory has demonstrated that the TC subunits of *P. luminescens* are post transcriptionally regulated, with the mRNA only being translated at the time of secretion [27]. Upon secretion the complex normally becomes associated with the outer surface of the cell. However some strains encode a small lipase in the *tcd* pathogenicity island (pai) [28], named Pdl, which enhances the secretion and facilitates the release of the TC into the surrounding milieu [27]. It remains obscure how the bacterium is able to secrete and assemble such a large multimeric protein complex onto the cell surface. A lack of recognisable export sequences has confounded an understanding of the mechanism of secretion.

**Figure 1:**
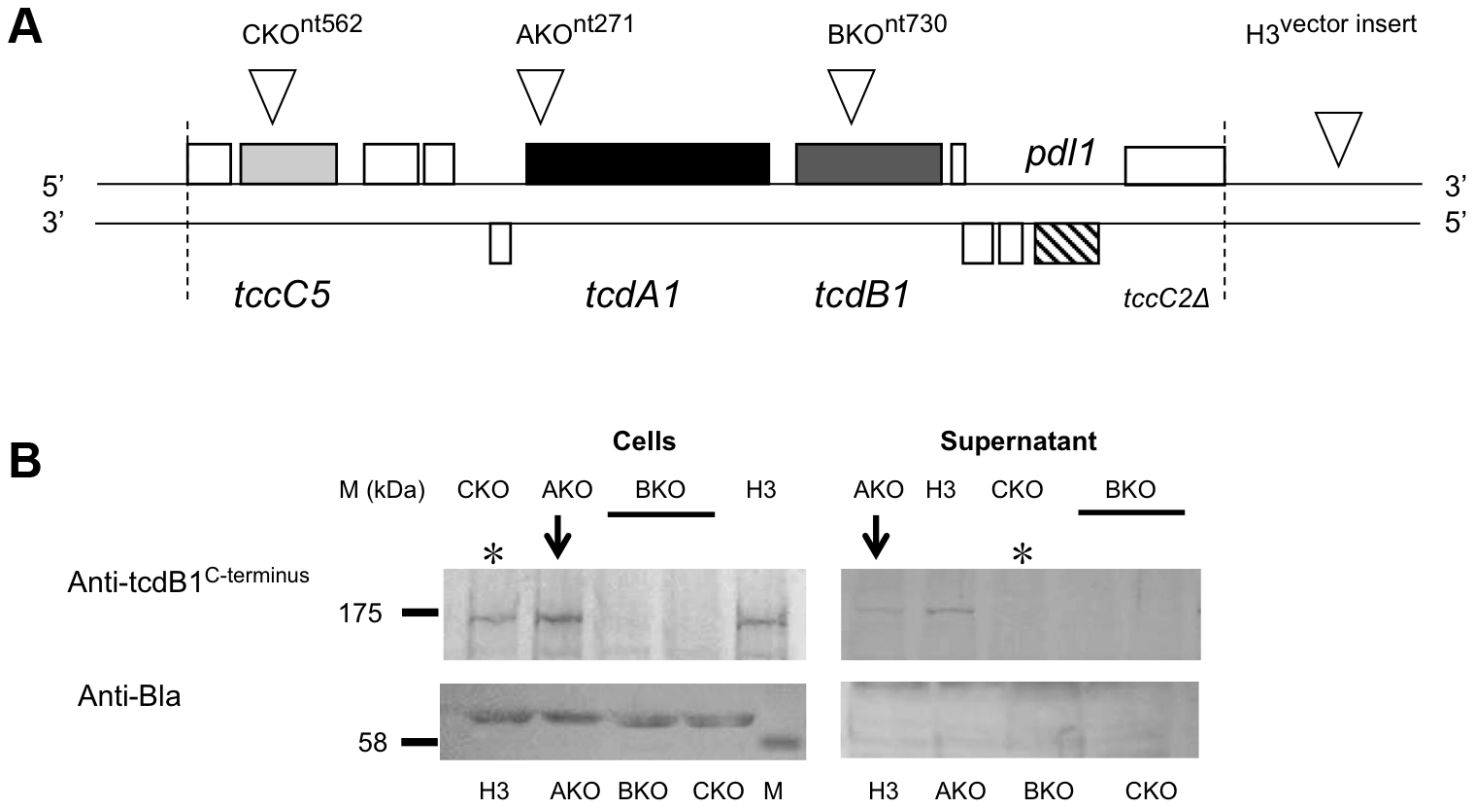
The cosmid model for heterologous TC synthesis and secretion in *E. coli*. (**A**) Map of cosmid (c1AH10) containing a portion of the *P. luminescens* W14 *tcd* pathogenicity island (bounded by dotted lines). Both strands are shown and the ORFs encoded on these are show as boxes. TC genes are shaded in grey and the *pdl1* release lipase gene is hatched [27]. Inverted triangles show the location of transposon insertion mutants. The AKO, BKO and CKO insertion points are at nucleotides 271, 730 and 562 respectively, with respect to the first nucleotides of the ORFs. Note “H3” represents a cosmid clone in which the transposon has inserted into the vector backbone outside of the insert and therefore contains the full intact functional locus. (**B**) Western blot analysis of whole cells (200 µl culture pellet) and concentrated supernatants (1.2 ml) from the cosmid model of various transposon insertion mutant clones. Supernatants are from 2 day old cultures grown at 28°C in LB medium. Antibodies used were raised against the C-terminus of the B-subunit, TcdB1. Two independent B1 KO clones where tested (bar). An antibody directed against β-lactamase (Anti-Bla) is used as a loading control for the quality of the soluble and membrane fractions in this and subsequent blots. Note that knock out (KO) of the C-subunit *tccC5* gene (CKO) prevents secretion of the B-subunit (*), while KO of the A-subunit *tcdA1* gene (AKO) does not prevent B-subunit secretion (arrow). Bioassays of these same samples can be seen in Figure S1.

Here we present an investigation into the functions of different domains of the B and C-subunit proteins. The large number of *tc* gene homologues in *Photorhabdus* makes the study of the export process in the original genus difficult. We therefore developed heterologous *E. coli* models to study the synthesis and export of the TC. We used both inducible expression systems and a cosmid model that utilises the native *Photorhabdus* expression signals [27]. The cosmid clone (Fig. 1A) encompasses the *tcd1A*, *tcdB1* and *tccC5* subunit genes from the *tcd* pai of *P. luminescens* strain W14 [28]. We have previously demonstrated that *E. coli* correctly synthesises and secretes an active Tcd complex from this cosmid with no loss of cell viability [27]. This confirms that all sequences required for synthesis and secretion are either associated with the *tc* genes themselves, or are common to the *Escherichia* genome.

2D-gel electrophoresis (Fig. S5) and previous heterologous expression studies [3], [12] indicate the B and C-subunits need to be produced together in the same cytoplasm in order to form a functional sub-complex. Here we demonstrate that the N-terminal domains of the B and C-subunit proteins, which are well conserved across family members, are both essential for secretion of the B+C sub-complex. We also confirm that while the C-terminal domain of the C-subunit is essential for toxicity, that it is irrelevant for secretion. Indeed, replacing the C-terminal domain with a FLAG-tag epitope reveals that it is possible to fuse alternative sequences to this gene and have it translated and exported. Interestingly an in-frame deletion of the B-subunit N-terminal domain strongly reduces production of both itself and a downstream C-subunit gene. These subunits can then no longer be detected in the supernatant. Despite this, the truncated-B+C sub-complex does still retain toxicity. We also show that the A-subunit is not required for the B+C sub-complex secretion. On the other hand we demonstrate that the A-subunit secretion (but not synthesis) *is* dependent upon the C-subunit. Even in the absence of the B-subunit, the C-subunit can facilitate secretion of the A-subunit. Using western blots we confirm that the C-subunit becomes localised to the periplasm and is unable to cross the outer membrane in the absence of a B-subunit. Furthermore, in the absence of a C-subunit, the B-subunit is unable to cross the inner membrane and remains associated with the cell spheroplast. Finally we demonstrate limited toxicity of the TccC5 C-subunit alone by injection into insects. We propose a model ascribing function to structure for the B and C-subunits based upon domain homologies and the experimental results presented here.

## Materials and Methods

### Construction of *E. coli* BL21 TC production strains

The *tcdA1*, *tcdB1* and *tccC5* genes were amplified from *P. luminescens* strain W14 genomic DNA using rTth DNA polymerase (Applied Biosystems). Polymerase chain reaction (PCR) conditions were 1.2–1.6 mM magnesium acetate, 2 mM each dNTP and 1 mM each primer. Thermocycling was performed as follows: 93°C for 30 s; 55°C for 30 s and 68°C for 8 min, for 30 cycles and final 68°C incubation for 10 min. PCR primers, used for cloning into the arabinose inducible expression vector pBAD30, were designed to include unique restriction sites for subsequent cloning. The primer sequences (5′ to 3′) used for cloning the *tcdA1*, *tcdB1* and N-terminally truncated *tcdB1* into pBAD30 were as follows:

1. **A (** ***tcdA1*** **):** A1_SacI_30F: AT*GAGCTC*GATTTAAAAGGAATAAATATG and A1_XbaI_30R: AT*TCTAGA*TTATTTAATGGTGTAGCGA.
2. **B (** ***tcdB1*** **):** B1_SacI_30F: AT*GAGCTC*GGGCCTGTAAGGAGTTTTTATGC and B1_XbaI_R: AT*TCTAGA*TTACACCAGCGCATCAGCGGCCGTATC. B1_FLAG_XbaI_R: ATTCTAGATTACTTGTCATCATCGTCCTTGTAGTC CACCAGCGCATCAGCGGCCGTATC.
3. **BΔC (** ***tcdB1*** **without N-terminus):** B1_SacI_1084F: AT*GAGCTC*GGGCCTGTAAGGAGTTTTTATGGACAACAACACGGTTACC and B1_XbaI_R: AT*TCTAGA*TTACACCAGCGCATCAGCGGCCGTATC.

For *tccC5* genes with a FLAG-tag, C5, C*sm* and C*osp* were first cloned into pFLAG-ctc to create FLAG-fusion genes. They were then subsequently PCR amplified from these templates for cloning into pBAD30 or pBAD30-*tcdB1* (downstream of the *tcdB1* gene). The primer sequences (5′ to 3′) were as follows; C5-30XbaI-f: AT*TCTAGA*AAGGAAGTAAATATGGAAAACATTGACCC; SphI_Flag_R: AT*GCATGC*CGATCGAGAGATCGATCTTCACTTGTCG.

1. **C5:** C5KpnI_for_Flag R: AT*GGTACC*ATTTGCACTGGATGATTTGAAATTACCGG.
2. **C** ***sm*** **:** C5sm2004KpnI_for_Flag R: AT*GGTACC*TCCCTGAACATCAAATTGCGTCACCGGG.
3. **C** ***osp*** **:** C5ospA1800KpnI_for_Flag R: AT*GGTACC*TGCCGCCCACAGCGCTGTACC. PCRs using Primer-C5-30XbaI-f and (1), (2) or (3) were used for cloning into pFLAG-ctc. PCRs using Primer-C5-30XbaI-f and SphI_Flag_R were used for cloning into pBAD30 or pBAD30-*tcdB1*.

For the N-terminal His-tag cloning of *tccC5* (pC5_N_his) the gene was initially cloned into pET28a to create a His-fusion before subsequent PCR amplification using this template to create an amplicon to clone into pBAD30. The primer sequences (5′ to 3′) were as follows: C5_NdeI_F: AT*CATATG*GAAAACATTGACCCAAAAC, C5_SphI_R: AT*GCATGC*TTAATTTGCACTGGATGA and pET28a_XbaI_F: CC*TCTAGA*AATAATTTTGTTTAACTTTAAGAAGGAGA. PCRs using primers C5_NdeI_F and C5_SphI_R were used for cloning into pET28a. PCRs using primers pET28a_XbaI _F and C5_SphI_R were used for cloning into pBAD30. For creation of the C-terminal His-tag fusions of *tccC5* (pC5_C_his and pC5*sm*_his), the full length C5 and truncated C5*sm* genes were cloned into pET28a to create His-fusions before subsequent PCR amplification using these templates to create amplicons for cloning into pBAD30. The primer sequences (5′ to 3′) were as follows: pET28a_SphI_R: AT*GCATGC*GCAGCCGGATCTCAGTGGT and **(1) C5**: C5_SacI_pET_R: AT*GAGCTC*ATTTGCACTGGATGATTTGAAATTACCGG and **(2) C5sm:** C5sm2004_SacI_pET_R: AT*GAGCTC*TCCCTGAACATCAAATTGCGTCACCGGG. PCRs using primers C5-30XbaI-f and (1) or (2) above were used for cloning into pET28a; PCRs using primers C5-30XbaI-f and pET28a_SphI_R were used for cloning into pBAD30.

Following PCR, amplicons were purified (using Millipore Montage PCR columns as per instructions), cut with the appropriate restriction enzyme(s), and then re-purified prior to ligation and cloning. Cloning vector DNA from pBAD30 or pFLAG-ctc was prepared (Qiagen miniprep kit as per instructions), with the relevant restriction enzyme(s). Ligations were performed at a 3∶1 molar excess of insert to vector using the Promega T4 DNA ligase rapid ligation system. Aliquots of the ligation reaction were electroporated into commercial pre-prepared EC100 *E. coli* cells (Epicentre) and recovered on Luria–Broth (LB) agar containing 100 µg/ml ampicillin. Correct constructs were selected by restriction digest of DNA prepared from candidate clones and verified by sequencing before transformation into *E. coli* BL21. Plasmid DNA from transformants were checked by restriction digest before storing the strains at −80°C in 15% glycerol-LB.

### Recombinant production of TC protein

Induction of expression strains was done as briefly described. Glycerol stocks were used to inoculate 5 ml of fresh LB media supplemented with 0.2% glucose (w/v) and the appropriate antibiotic for selection. Bacteria were grown overnight at 28°C with aeration, 1 ml of this culture was then harvested and resuspended in 100 ml of the same media and incubated at 28°C until an OD_600_ of 0.7–0.9 was achieved. Cells were then harvested at room temperature by 10 min centrifugation at 4000 rpm. The pellet was re-suspended in 100 ml of fresh LB, supplemented with the appropriate antibiotic and 0.2% (w/v) of the pBAD30 promoter (P*ara*) inducer, L-arabinose. Cells or trichloroacetic acid (TCA) precipitated and concentrated supernatants from 3 hour induced cultures were collected for analysis using SDS-PAGE and western blot to confirm synthesis.

### *Manduca sexta* oral toxicity bioassays

Supernatants or cell extract samples were diluted in 1× phosphate-buffered saline (PBS) and applied to 1 cm^3^ disks of artificial wheat germ diet as previously described [22]. Treated food blocks were allowed to dry for 30 min and two neonate *M. sexta* larvae were placed on each food block before incubation at 25°C. Larvae were allowed to feed for 7 days before being weighed. Growth differences are then expressed as mean larval weight (MLW) in grams. Note there was no mortality incurred in these assays as discriminatory toxin dilutions were used.

### Complementation bioassays

*E. coli* strains containing the required cosmids (various *tc* gene insertional knock outs) were grown for two days at 28°C with aeration. Cells were removed from culture supernatants by centrifugation and passed through a 0.22 µm filter (Millipore). Cell extract samples from heterologous production strains were induced for 3 hours before harvesting by centrifugation, washing in 1×PBS and lysis by sonication. Cell debris and unbroken cells were removed by low speed centrifugation. Samples were mixed at the desired ratio at room temperature for 30 min, diluted as indicated, and then applied to artificial food disks as described for the *Manduca sexta* oral toxicity bioassay method.

### Cell fractionation

Heterologous production strains were induced for 3 hours. Cells were washed in 1×PBS, normalized to an OD600 optical density of 10.0 before lysis using sonication (10 s on and 10 s off, 45% power regime for 3 min). Unbroken cells and debris were removed by low speed centrifugation. Cell-free lysates were fractionated into soluble and insoluble sub-fractions by ultracentrifugation (28 000 r.p.m. for 2 h in a Beckman SW40Ti rotor). The insoluble fraction was resuspended in 1×PBS to restore its original concentration relative to the soluble fraction proteins in the lysate. Supernatant samples were prepared and concentrated using TCA precipitation. The spheroplast and periplasmic fractions were isolated using a Periplasting Kit as described by the manufacturer (Epicentre Biotechnologies).

### Western blot

Protein fractions were separated by 1 dimensional SDS-PAGE and western blotted onto Nitrocellulose using a semi-dry blotter (Biorad). We probed these membranes using a standard protocol with the following antibodies: TcdB1^C-terminus^ anti-peptide antibody (raised against aa856-YSSSEEKPFSPPND*C*-aa869); TccC1^N-terminus^ anti-peptide antibody (raised against aa347-FWRNQKVEPENRYV*C*-aa360), a monoclonal anti-FLAG antibody (SIGMA) and monoclonal antibodies against the RNA polymerase andβ-lactamase (Abcam). Immune-reactive bands were visualized using alkaline phosphatase-conjugated anti-rabbit secondary antibody (SIGMA) at 1∶5000 and developed using a NBT-BCIP reagent.

### Accession numbers

NCBI accession numbers for the proteins described in this study are as follows: TcdA1, AAL18486; TcdB1, AAL18487; TccC5, AAO17210.

## Results

### The N-terminal domain of the C-subunit is essential for the secretion of the B-subunit

An *E. coli* cosmid clone model was previously characterised for the study of TC secretion at native expression levels [27]. From hereon we will use the terms A-, B- and C-subunits to refer specifically to the TcdA1, TcdB1 and TccC5 proteins encoded on this cosmid. We used transposon insertions to determine the relevance of the A and C-subunit genes on secretion of the B-subunit (Fig. 1A). The transposon insertion points were chosen so as to abolish the majority of each gene. As expected when any of the three subunit genes were knocked out (KO) by transposon insertion, then oral toxicity of the supernatant was lost (Fig. S1). Western blot analysis of these supernatants revealed that KO of the C-subunit prevented secretion of the B-subunit, with all the protein remaining in the whole cell samples (Fig. 1B*). KO of the A-subunit gene did not prevent B-subunit secretion into the supernatant (Fig. 1B-arrows). Note detection of TcdB1 synthesis in the AKO cosmid clone confirms there is no polar effect caused by the transposon insertion.

**Figure 2:**
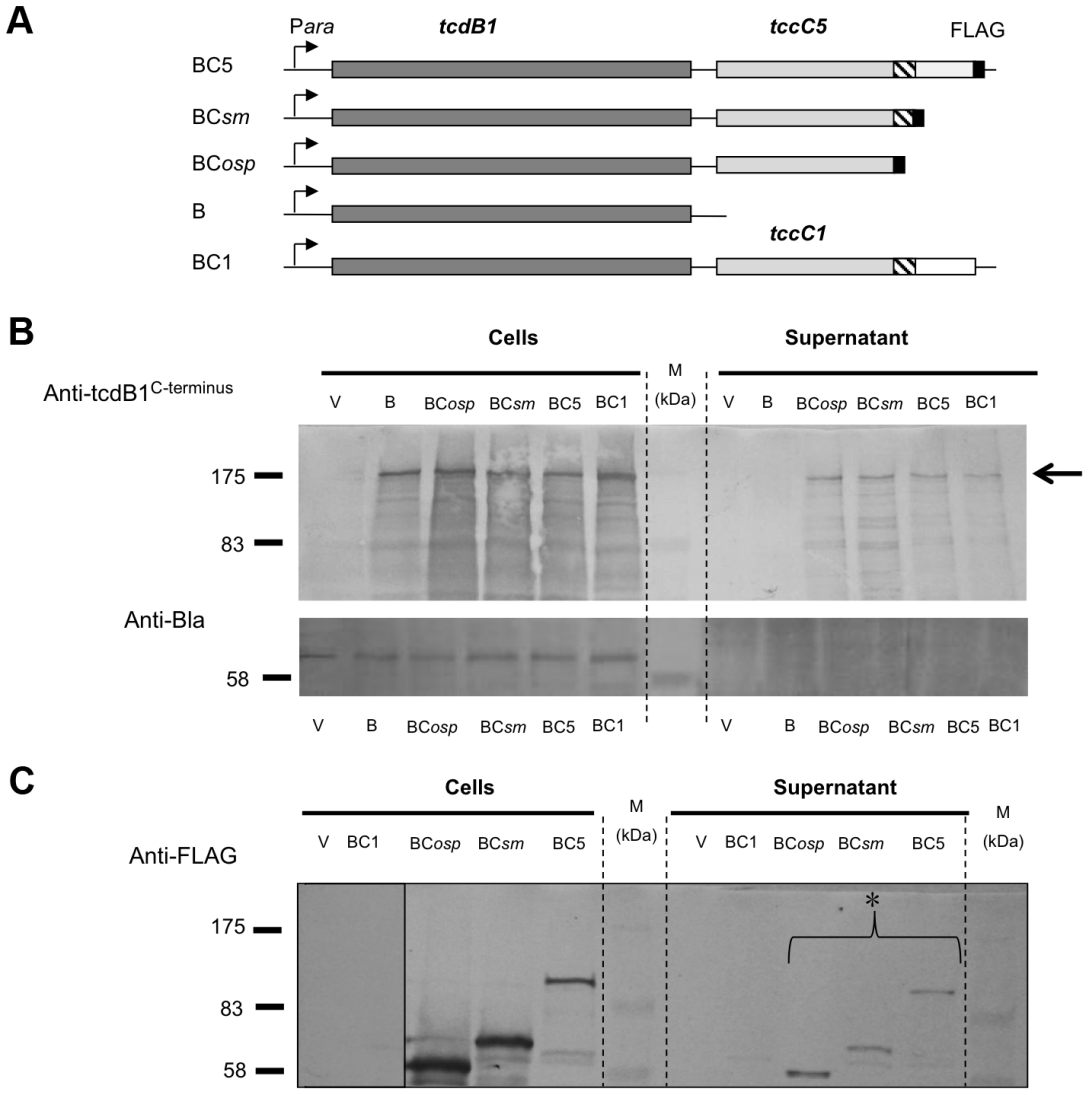
The N-terminus of the C-subunit is essential for secretion of the B+C sub-complex. (**A**) Arabinose inducible (P*ara*) *E. coli* heterologous B+C sub-complex expression constructs (in pBAD30) containing an intact copy of *tcdB1* and different modified *tccC* genes. Native translation initiation regions were used. The *tccC5* genes each contain a C-terminal FLAG tag at different locations and are designated; BC5, BC*sm* and BC*osp*. The hatched region on the *tccC* genes represents a region highly conserved in “Rhs/C-subunit” family proteins. Construct “BC5” encodes the full length of *tccC5*; “BC*sm*” encodes the first 668 amino acids of TccC5 and “BC*osp*” the first 600 amino acids of TccC5. “B” represents a *tcdB1* only construct and “BC1” represents a construct for co-expression of the *tcdB1* and *tccC1* genes [22]. The “V” negative control samples are from *E. coli* harbouring the empty expression plasmid pBAD30 only. (**B**) Western blot analysis of cells and concentrated supernatants of these constructs using an anti-peptide antibody raised against the C-terminus of B-subunit, TcdB1. The arrow indicator shows the secreted TcdB1. An anti β-lactamase (Anti-Bla) Western blot is included to provide a control for sample quality and loading amount. (**C**) Western blot analysis of these same cell and concentrated supernatant samples using an anti-FLAG tag antibody to determine the location of the tagged truncated C-subunit proteins, as indicated by the asterisk.

We also constructed a series of inducible expression strains containing the B-subunit gene linked to different truncated versions of C-subunit genes. These truncations had FLAG tags fused at their C-termini to allow detection by western blot (Fig. 2A). We used these constructs to assess the importance of the C-terminal domain of the C-subunit upon secretion of the B+C sub-complex. Figure 2B (arrow) illustrates that the N-terminus of the C-subunit (aa1–600) is necessary and sufficient for the secretion of the full length B-subunit. Detection of the FLAG-epitopes in these fusions shows that the C-subunit is also being exported (Fig. 2C*). This also demonstrates that it is possible to export non-TC protein sequences fused downstream of aa600 of the C-subunit without hindering the export process. While this data confirms that the C-terminus of the C-subunit is not relevant to secretion, complementation bioassays (see below) using these constructs confirm that it is necessary for toxicity as expected (Fig. S2).

### The A-subunit secretion but not synthesis is also dependent upon the C-subunit

**Figure 3:**
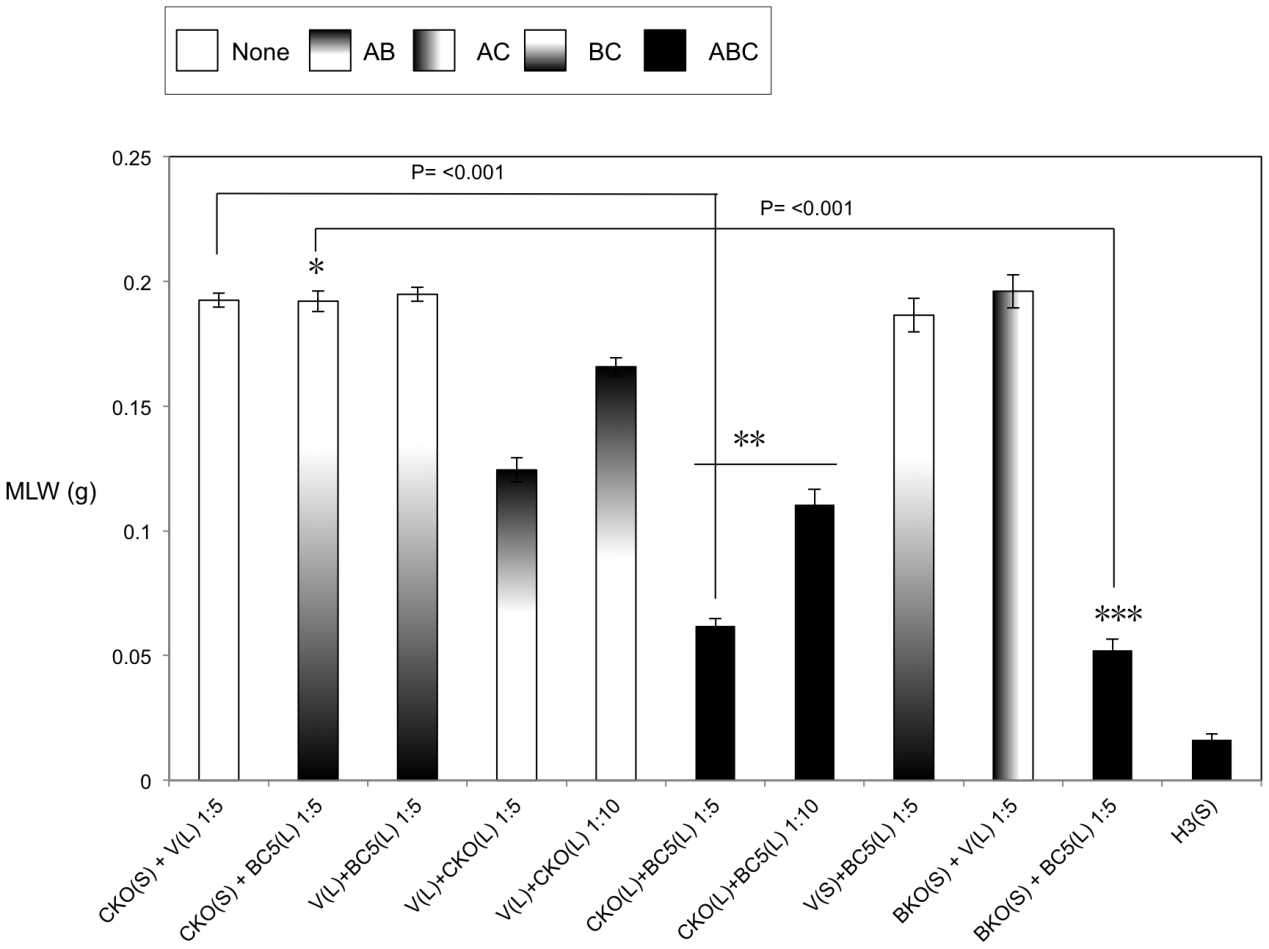
Cosmid complementation bioassay showing the A-subunit secretion but not synthesis is dependent upon the C-subunit. Mean larval weight (MLW) gain of cohorts of *M. sexta* neonates (n = 10) fed dilutions of mixed sonicated cell lysates (L) and culture supernatants (S), at dilutions of either 1∶5 or 1∶10, from the cosmid model and other bespoke heterologous production strains. Samples were mixed at a ratio of 50 µl of lysate (L) to 950 µl of supernatants (S). Error bars represent the standard error. The construct designations are consistent with those in figure 2. The H3, CKO and BKO cosmid insertional knock out variants are as shown in figure 1. Note, the more potent the toxic effect, the smaller the mean larval weight. A key using the data column fill pattern is given above the graph to assist in the interpretation of the predicted TC subunit contents of the test samples. The *, ** and *** indicators are discussed in text. Two sample *t*-test comparisons were used to confirm statistical significance in mean weight differences at 99% confidence between the following samples discussed in the text; [CKO(S)+BC5(L) 1∶5] vs. [CKO(L)+BC5(L) 1∶5]: t = 25.72, P<0.001, df = 18. The [CKO(S)+BC5(L) 1∶5] vs. [BKO(S)+BC5(L) 1∶5] t = 22.54, P<0.001, df = 18.

**Figure 4:**
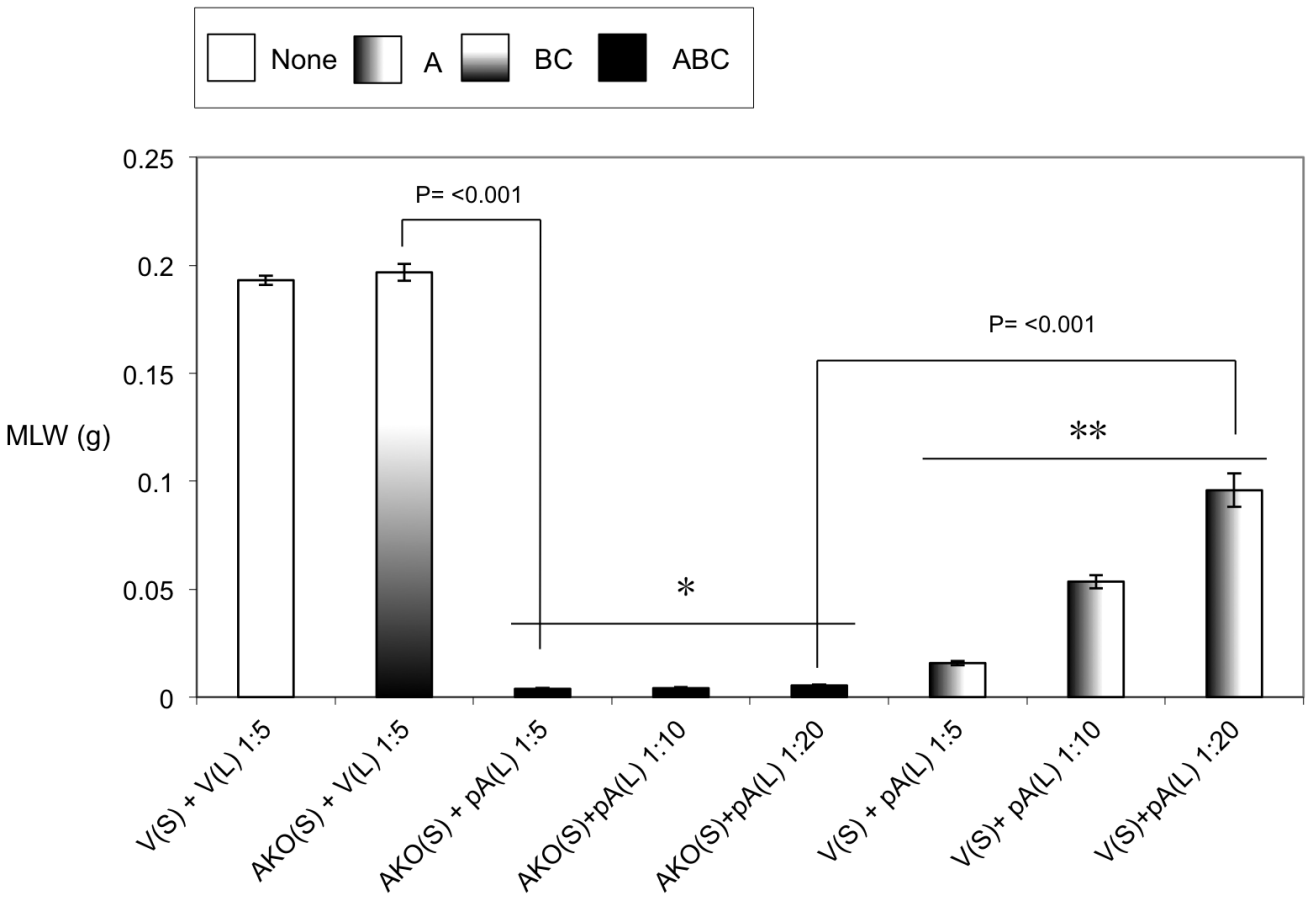
Cosmid complementation bioassay showing the B+C sub-complex secretion is independent of the A-subunit. Mean larval weight (MLW) gain of cohorts of *M. sexta* neonates (n = 10) fed mixtures of sonicated cell extracts (L) and culture supernatants (S), at dilutions of 1∶5, 1∶10 or 1∶20. Samples were mixed at a ratio of 50 µl of lysate (L) to 950 µl of supernatants (S). Samples were taken from the cosmid mutant AKO (figure 1) and pBAD30 expression constructs containing either nothing, “V”, or the *tcdA1* gene only, “pA” [22]. Error bars represent the standard error. The more potent the toxic effect, the smaller the mean larval weight. A key using the data column fill pattern is given above the graph to assist in the interpretation of the predicted TC subunit contents of the test samples. The indicators * and ** are discussed in text. Two sample *t*-test comparisons were used to confirm statistical significance in mean weight differences at 99% confidence between the following samples discussed in the text; [AKO(S)+V(L) 1∶5] vs. [AKO(S)+pA(L) 1∶5]: t = 47.75, P<0.001, df = 18 and [AKO(S)+pA(L) 1∶20] vs. [V(S)+pA(L) 1∶20]: t = 11.84, P<0.001, df = 18.

Previous studies have demonstrated that it is possible to mix A-subunits and the B+C sub-complex post-translation and recover the full toxic potential of the whole TC [22]. In the absence of an anti A-subunit antibody, we were able to use this observation to devise an oral toxicity “complementation bioassay” which we could use to determine the location and activity of different toxin subunits. This assay relies upon mixing sonicated cell extracts and culture supernatants from the cosmid model and bespoke heterologous production strains and then testing toxicity levels by feeding to *Manduca sexta* neonate larvae. Using these assays we were able to reveal the impact that the three different subunits have upon the synthesis and secretion of one another. Figure 3 demonstrates that when the C-subunit gene is inactivated (CKO), that we can no longer detect bioactive A-subunit protein in the supernatants using the complementation bioassays. This is reflected as a lack of the ability of the CKO supernatant to complement the activity of B+C sub-complex (Fig. 3*). Note this effect is specific to a defect in the secretion of the A-subunit not its synthesis. This is shown by the ability of sonicated cell extracts from this same CKO strain to complement the B+C sub-complex (Fig. 3**). These experiments also show that the C-subunit can facilitate the secretion of the A-subunit in the cosmid model even when the B-subunit gene has been inactivated (BKO) (Fig. 3***). As expected these data also confirm (from several combinations shown) that while all three subunits are required for full toxicity, that some limited toxicity can be seen from the A-subunit alone when released from the cell by sonication. Figure 4 illustrates that when we mix supernatants from a cosmid clone in which the A-subunit is knocked out (AKO) with A-subunit protein released by sonication from cells of the A-subunit production strain (pA), that we again reconstitute a fully toxic TC (Fig. 4*). Note the oral toxicity of the A-subunit protein alone when released from the pA production strain by sonication is significantly lower (Fig. 4**). This confirms that B+C sub-complex secretion from the cosmid clone is not dependent upon the presence of a functional A-subunit.

### The B-subunit is required for C-subunit secretion

**Figure 5:**
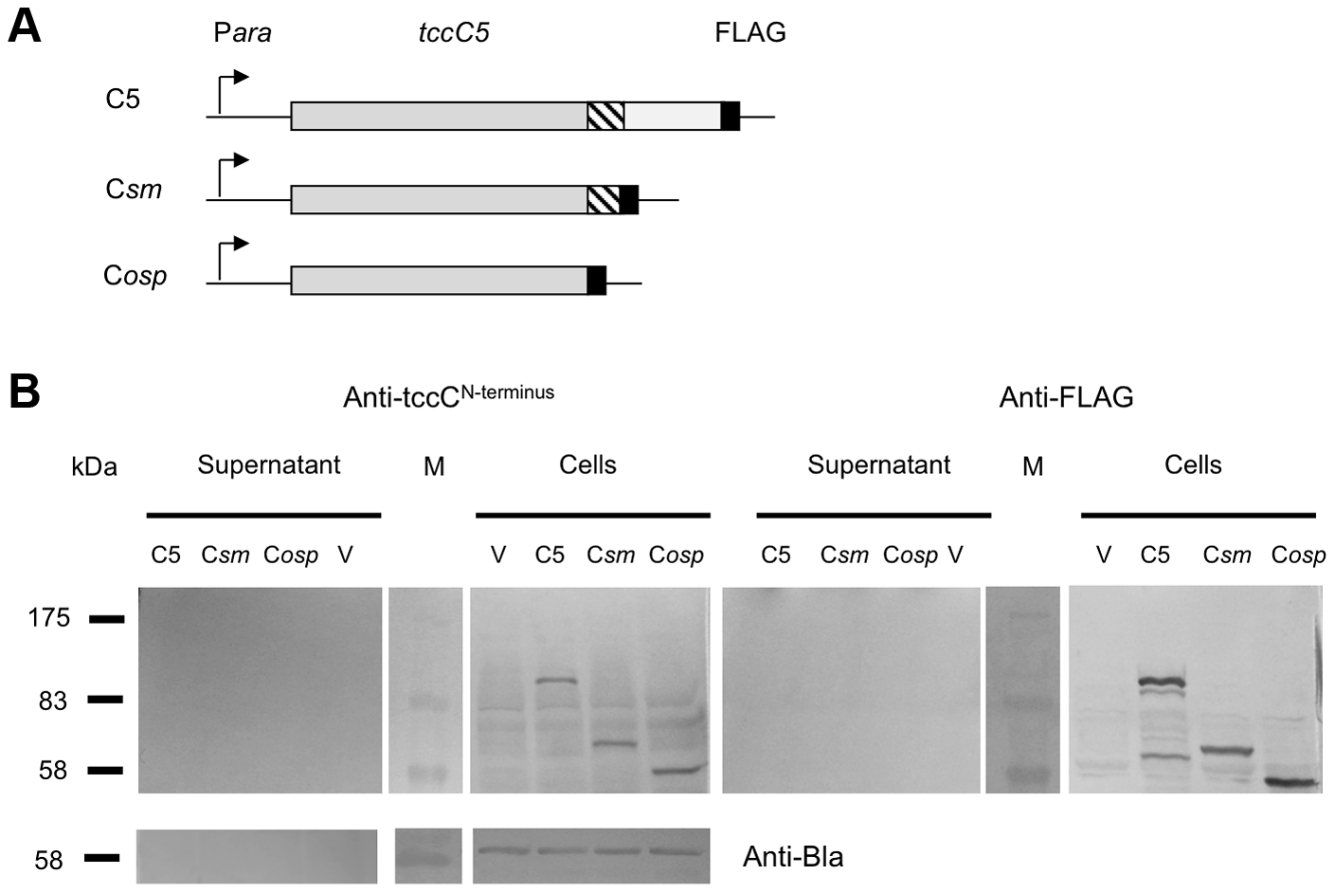
C-subunit secretion requires the B-subunit. (**A**) Arabinose inducible *E. coli* heterologous *tccC5* expression constructions in pBAD30. The *tccC5* genes each contain a C-terminal FLAG tag at different locations and are designated; C5, C*sm* and C*osp*. Native translation initiation regions were used. Construct “C5” encodes the full length of *tccC5*; “C*sm*” encodes the first 668 amino acids of TccC5 and “C*osp*” the first 600 amino acids of TccC5. (**B**) Western blot analysis of the cells and concentrated supernatants of these induced constructs using both an anti-peptide antibody raised against the N-terminus of TccC and an anti-FLAG tag antibody. An anti β-lactamase (Anti-Bla) Western blot is included to provide a control for sample quality and loading amount. Note despite good intracellular synthesis levels, no C-subunit protein could be detected in supernatant in the absence of the B-subunit.

**Figure 6:**
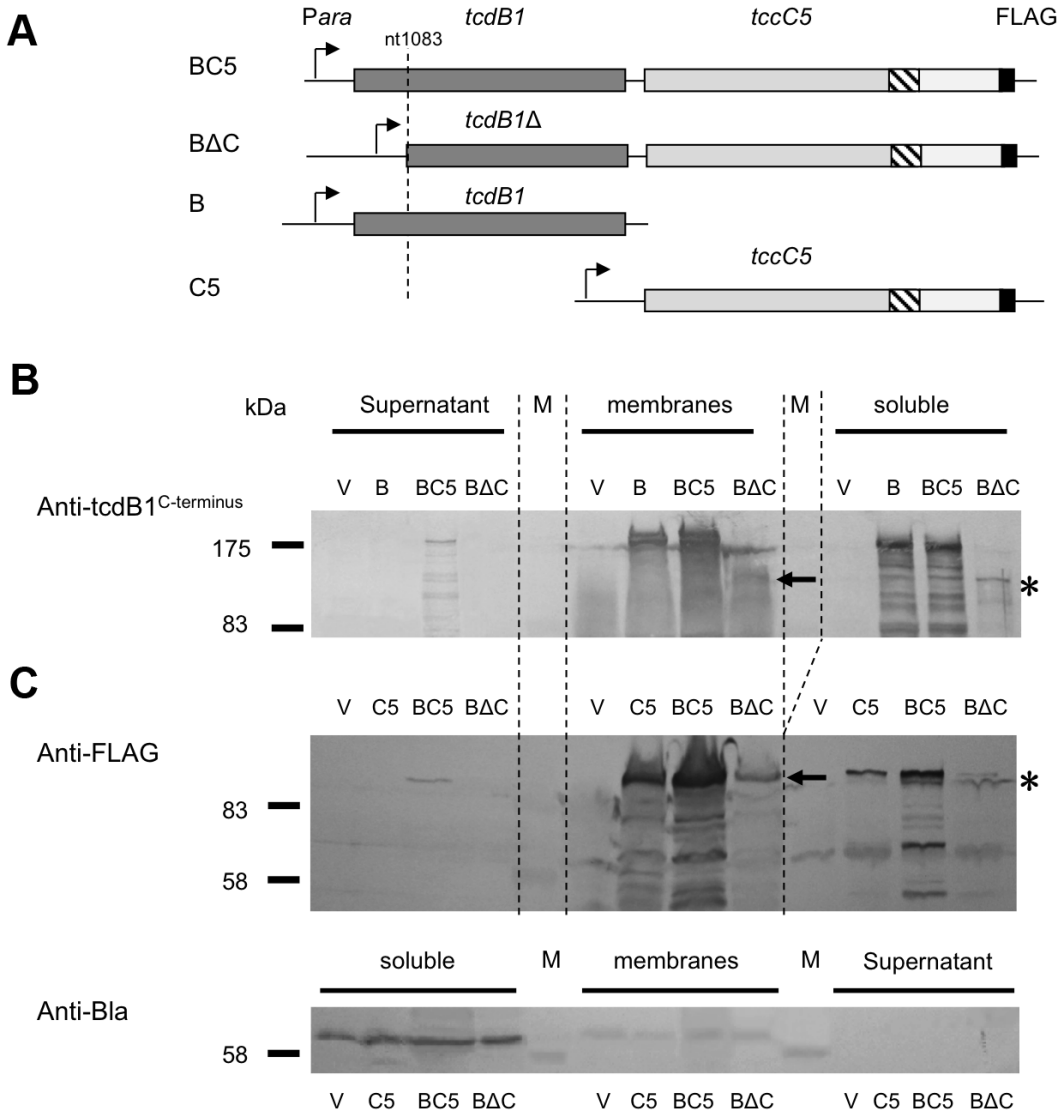
The N-terminus of the B-subunit is required for normal B+C sub-complex synthesis and secretion. (**A**) Arabinose inducible *E. coli* heterologous production constructions encoding full length or N-terminal truncated *tcdB1* with *tccC5* containing C-terminal FLAG-tag fusions. Construct “BC5” encodes full-length *tcdB1* and tagged *tccC5*, “BΔC” encodes an N-terminal truncated copy of *tcdB1* and a tagged *tccC5*, “B” encodes full-length *tcdB1* only and “C5” encodes the tagged *tccC5* only. “V” represents pBAD30 vector only control. All constructs use the native translation initiation regions. (**B**) Western blot analysis of concentrated supernatants, membrane (inner and outer) and soluble fractions (cytoplasm and periplasmic contents) of these induced constructs using an anti-peptide antibody that was raised against the C-terminus of B-subunit. (**C**) Western blot analysis of these same samples using an anti-FLAG tag antibody. The asterisk and arrow indicators are discussed in the text. An anti β-lactamase (Anti-Bla) Western blot is included to provide a control for fractionation sample quality and loading amount.

**Figure 7:**
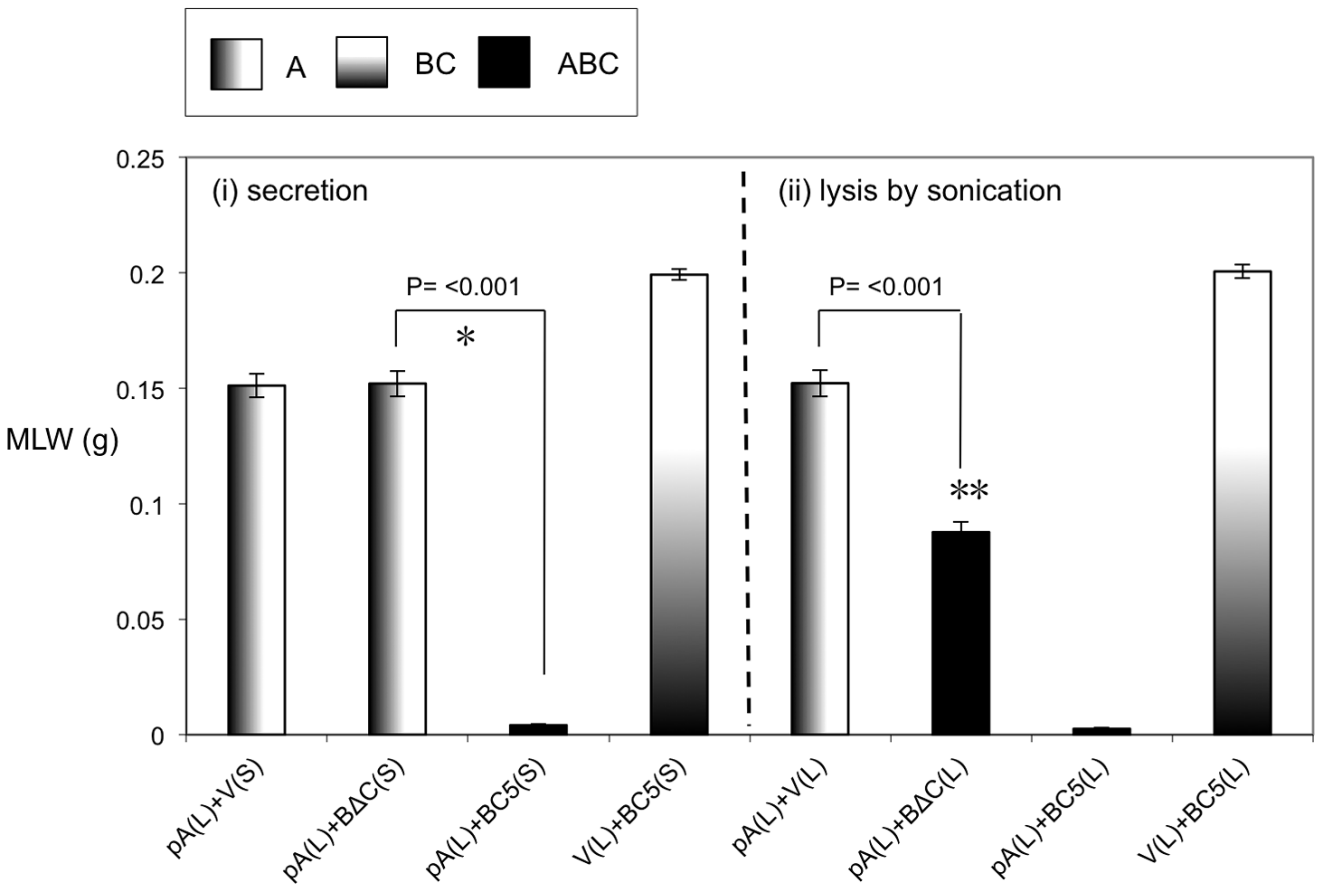
The N-terminal 361 amino acids of TcdB1 are not essential for toxicity. Mean larval weight (MLW) gain of cohorts of *M. sexta* neonates (n = 10) fed 100 µl of mixtures of sonicated cell extracts (L) and culture supernatants (S). Arabinose inducible expression constructs tested include the pBAD30 vector only, “V”, the *tcdA1* only construct, “pA” (figure 4) and the BC5 and BΔC constructs (figure 6). Sample mixes were as either (i) 50 µl lysed pA sample complemented with 950 µl induced supernatant (left panel) or (ii) 50 µl lysed pA sample complemented with 50 µl lysed test sample and diluted with 900 µl of PBS. Error bars represent the standard error. The more potent the toxic effect, the smaller the mean larval weight. A key using the data column fill pattern is given above the graph to assist in the interpretation of the predicted TC subunit contents of the test samples. The indicators * and ** are discussed in text. Two sample *t*-test comparisons were used to confirm statistical significance in mean weight differences at 99% confidence between the following samples discussed in the text; [pA(L)+BΔC(S)] vs. [pA(L)+BC5(S)]: t = 26.68, P<0.001, df = 18. [pA(L)+V(L)] vs. [pA(L)+BΔC(L)]: t = 8.95, P<0.001, df = 18.

We heterologously expressed truncated and C-terminal FLAG-tagged versions of the C-subunit in *E. coli* (Fig. 5A). Western blots using an anti-C-subunit N-terminal anti-peptide antibody and an anti-FLAG antibody to detect the C-terminal tags confirmed that the C-subunit could not be secreted in the absence of the B-subunit (Fig. 5B). This is in contrast to the results presented in figure 2 showing that when the B and C-subunits are co-expressed, that they are both secreted. We also expressed intact or N-terminally truncated copies of the B-subunit gene (removing aa1–361) as bi-cistrons with C-terminal FLAG tagged copies of the full length C-subunit gene (Fig. 6A). We used Western blots with anti-TcdB1 C-terminus and FLAG tag antibodies to determine the effect of the N-terminal truncation of the B-subunit on the secretion of the B+C sub-complex. The secretion of individually expressed copies of the B- and C-subunit genes was also examined in this way. Figure 6 shows that removal of the first 361 amino acids of the B-subunit results in a failure to secrete either the B or C-subunits into the supernatant (Fig. 6B). It should be noted however that removal of these amino acids from the B-subunit also significantly reduced the relative synthesis levels both of itself and of the downstream C-subunit seen in the soluble cell fractions (cytoplasm and periplasm) (Fig. 6*). The amount of B+C sub-complex seen in the membrane fraction was also reduced (Fig. 6 arrows). It is not clear whether removal of the N-terminus of the B-subunit causes a complete abolition of secretion or whether we are failing to detect the lower levels of the B+C sub-complex that is secreted by these strains. Nevertheless, the importance of this region to the production level of the B+C sub-complex is clear. Complementation bioassays using supernatants from these strains confirmed that they showed a loss of toxicity (Fig. 7*). Despite the drop in B and C-subunit production levels, this “truncated” sub-complex was still functional, able to show complementation with the A-subunit when released from the cell by sonication (Fig. 7**). This indicates that the N-terminal 361 aa of the B-subunit are not essential for toxicity, although they do influence the synthesis and export levels of the B+C sub-complex.

### Localisation of the B and C subunit proteins when expressed independently

**Figure 8:**
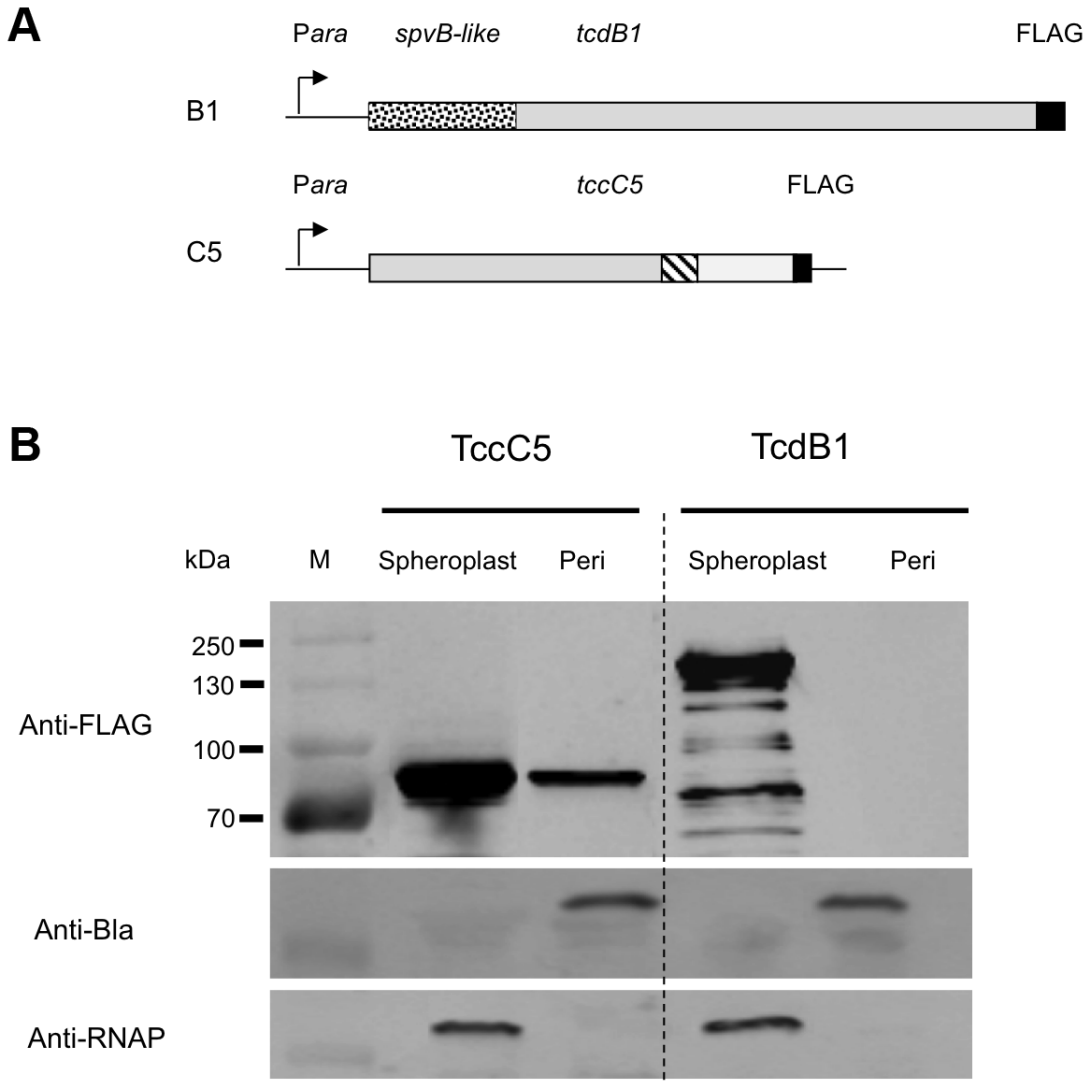
Localisation of the B and C subunits by fractionation and western blotting. (**A**) The *tcdB1* and *tccC5* genes were PCR cloned as C-terminal FLAG-tag epitope fusions into the arabinose inducible expression plasmid pBAD30 in *E. coli*. The *spvB-like*, *rhs* core-like and toxin domains are shown as dotted, hatched and open boxed regions respectively. FLAG-tags are shown as filled boxes. (**B**) Induced cells were separated into spheroplast and periplasmic (peri) protein fractions. Western blots using the anti-FLAG antibody were then used to identify the location of these two subunits. Anti-β-lactamase (Anti-Bla) and anti-cytoplasmic RNA polymerase (Anti-RNAP) western blots were performed on these samples to provide controls for sample quality and loading amount. Note TccC5 was detected in the periplasmic fraction, while TcdB1 was restricted to the spheroplasts.

We PCR cloned the *tcdB1* and *tccC5* genes as C-terminal FLAG-tag epitope fusions into the arabinose inducible expression plasmid pBAD30 in *E. coli* (Fig. 8A). Induced cells were then separated into spheroplast and periplasmic fractions. Western blots using the anti-FLAG antibody were then used to identify the location of these two subunits (Fig. 8B). While we were able to detect the C-subunit in the periplasmic fraction, the B-subunit was restricted to the cell spheroplasts. This confirms that the N-terminus of the C-subunit is able to direct its secretion across the inner membrane and that the B-subunit N-terminus is then required to facilitate secretion, of the whole B+C-sub-complex across the outer membrane. This also demonstrates that the B-subunit requires the presence of the C-subunit in order to cross the inner membrane.

### Toxicity of truncated and intact C-subunit protein

Over-production of C-terminally truncated copies of the C-subunit (Fig. 5A) in the original *P. luminescens* W14 strain led to a significant reduction in the oral toxicity of the culture supernatant to *M. sexta* neonates (Fig. S3). This showed that the non-toxic overexpressed N-terminal portion (aa1–600) of the C-subunit protein is able to interfere with either synthesis or secretion. Finally we tested the toxicity of full length and truncated heterologously expressed C-subunit proteins by injection into *Galleria* larvae. For these experiments we constructed N- and C-terminally His-tagged expression constructs and a tagged C-terminally truncated version of the gene (Fig. S4A). When we injected sonicated cell samples of these production strains into *Galleria* it demonstrated that the full length C-subunit could show some level of toxicity independent of the A- and B-subunits (Fig. S4B). As expected, the C-terminally truncated constructs showed no toxicity again confirming the C-terminal tail as the toxin encoding domain.

## Discussion

Our previous studies in *P. luminescens* W14 showed that abundant *tc* mRNA is constitutively transcribed for all the three *tcd* subunits, during laboratory culture in rich medium [27]. Nevertheless, Western blot analysis and oral toxicity bioassays confirmed that they are not translated until stationary phase at a time concurrent with secretion into the surrounding milieu [27]. This suggests that translation of the TC is tightly linked to export. Previous publications and our own experimental evidence (Fig. S5) show that the TC toxin B and C -subunits become tightly bound to one another in the bacterial cytoplasm, at least during heterologous co-expression in *E. coli* [3], [12]. In addition, when over-expressed together in *E. coli* BL21, TcdB1 and TccC1 can show limited toxicity to *M. sexta* even in the absence of an A-subunit [3]. Conversely, when synthesised in separate cells, released by sonication and mixed together post-synthesis no toxicity is observed [3]. This suggests that these two proteins may either associate during the translation process or require chaperone activity to assemble together. We note that homology detection and structure predictions using HHpred [29] show that that C-terminus of the B-subunit (aa1150–1450) and N-terminus of the C-subunit (aa20–380) both contain OspA-like domains (C5: P = 99.96 E-value = 1.9e-26; B1: P = 99.90 E-value = 7.7e-20). This structural domain in the OspA protein of *Borrelia burgdorferi* is responsible for homo-dimerization [30]. We suggest that these domains are responsible for the association of the B and C -subunits in the cytoplasm. Interestingly, although both these regions show high structural similarity to the OspA domain, there is no identity between them. It is tempting to speculate that this may represent a mechanism to prevent homo-dimerization of the subunits, and drive the association of the B and C -subunits in a 1∶1 ratio.

Examination of DNA databases reveals that genetic fusions of B and C –subunit gene homologues are encoded by a range of other pathogens, including the RhsT toxin of *Psuedomonas* and the YP_335336 protein of *Burkholderia pseudomallei* 1710b. We note that these large fusions only possess a single predicted OspA like domains (e.g. aa1400–1800 in YP_335336).

The work presented here demonstrates that the N-termini of both the B and C -subunits are required for secretion of all three subunits of Tcd. Furthermore, neither the B nor C -subunits can be secreted when expressed independently indicating that they both contribute to the process. Localisation experiments confirmed that the N-terminus of the C-subunit is required for secretion across the inner membrane and that the N-terminus of the B-subunit is responsible for secretion across the outer membrane. Interestingly we observed that the C-subunit alone could enable the secretion of the A-subunit. However, we saw no evidence of the secretion of the A-subunit by B in the absence of C. Our truncated and tagged B+C synthesis constructs also confirmed that the C-terminal domain of the C-subunit is not required for secretion. This is consistent with previous publications that this domain encodes the actual toxin [14], which is variable among homologues [2]. Interestingly, in our studies (data not shown) and in previously published work [12], there is evidence that at least in some cases, the C-terminal domain of C-subunit proteins may be cleaved and is not covalently attached to the rest of the TC complex. The C-subunit proteins belong to a large and enigmatic family of proteins called the Rhs-family and previous studies have also implicated Rhs proteins in export processes in *E. coli* and *Pseudomonas* [31], [32]. The large RhsT protein of *Pseudomonas aeruginosa*, which has recently been recognised as a secreted mammalian virulence factor [33], can also be seen to contain domains homologous to both the B and C-subunit proteins. Although the authors did not suggest a secretion method for this protein, it also has a toxic C-terminal domain, which in that case can be cleaved off and enter the host cell.

The normal route of TC delivery by *Photorhabdus* is direct release into the insect blood. It is interesting that we were able to show limited toxicity of the TccC5 C-subunit alone when overexpressed, released by sonication and injected directly into *Galleria* larvae. This effect was abolished by removal of the toxin encoding tail as expected. This nevertheless suggests that the C-subunit can gain entry into host cells independently of the other components of the TC. We speculate that the export mechanism of the C-subunit might actually be acting as a host cell import mechanism in this case.

**Figure 9:**
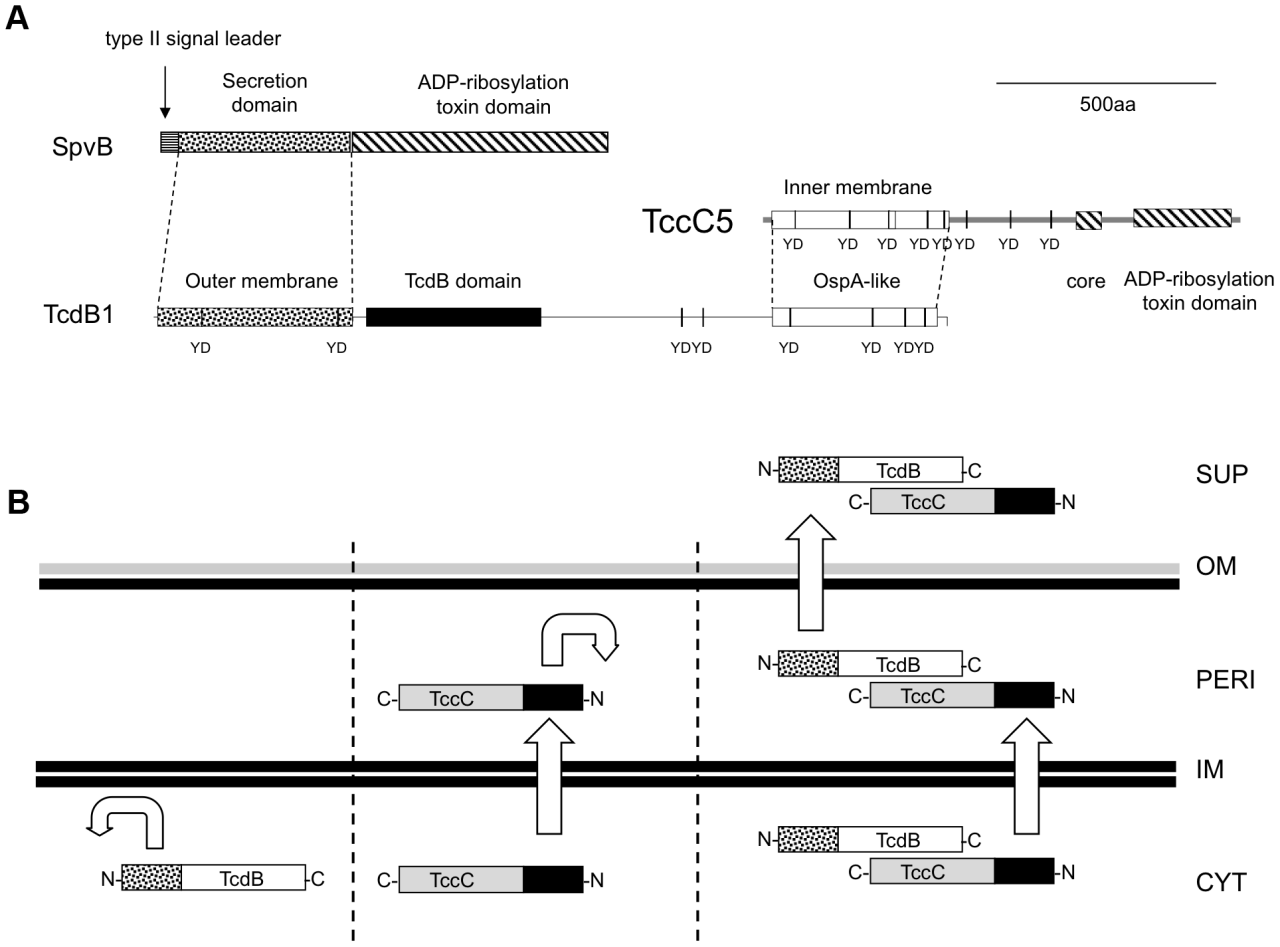
The predicted domain structure and function of B and C-subunits. (**A**) Comparisons of domain predictions of B+C sub-complex proteins and the *Salmonella* SpvB protein. Note, neither the B nor C -subunits contain a predicted type II signal leader present in SpvB. Note the “core” domain represents a highly conserved sequence common to all Rhs/TccC family proteins located adjacent to the variable C-terminal regions. The predicted OspA-like structural domains of the B and C proteins are shown in addition to YD-repeat sequences common to these protein families. The labels “inner membrane” and “outer membrane” on the N-terminal domains of the C and B -subunits reflect their secretion roles. (**B**) A diagrammatic summary of the fates of B and C-subunit proteins when produced either independently or together. Cellular compartments shown include supernatant (SUP), outer membrane (OM), periplasm (PERI), inner membrane (IM) and cytoplasm (CYT). The C and N-termini of the TcdB and TccC proteins are indicated. The straight and curved arrows show the ability (or not) of N-terminal domains to facilitate aspects of the export process.

The similarity of the B-subunit N-terminus to that of the SpvB protein of *Salmonella* suggests that this domain represents a secretion device, as it does in SpvB [18]. In SpvB, the N-terminal 1–229 amino acids are sufficient to promote secretion into extracellular milieu of the whole protein [18]. We note however that unlike SpvB, the B-subunit proteins do not possess a general sec-pathway type II signal leader (Fig. 9A). Our evidence suggests that the SpvB-like 361 N-terminal amino acids of the B-subunit is responsible for crossing the outer membrane only. Interestingly, when transiently expressed in mammalian cells, the N-terminus of TcdB1 was seen to accumulate in the nucleus [3]. We previously proposed that this region might represent a nuclear-targeting domain involved in toxicity to the host cell. However an alternative explanation is that this nuclear accumulation reflects a “one-way” membrane crossing activity of this N-terminal domain, which is normally used in secretion of Tcd across the Gram-negative outer membrane. Additional roles in crossing host cell membranes are not excluded by a role in secretion.

In previous work we used anti-TcdB1^N-terminus^ and anti-TcdB1^C-terminus^ antibodies to examine the translation and export of the B-subunit in *P. luminescens* W14 and the *E. coli* cosmid model [27]. Consistent with the manifestation of oral toxicity, TcdB1 can be detected in culture supernatants after 1 day, but not earlier when using the anti-TcdB1^C-terminus^ antibody. Conversely we observed only minor amounts of the TcdB1 in the supernatant when we used the anti-TcdB^N-terminus^ antibody [27]. This suggests that the N-terminus may be cleaved off during the secretion process as the C and N terminal antibodies show similar detection efficacy against intracellular TcdB1 protein. In addition when using the anti-TcdB1^C-terminus^ antibody, TcdB1 could be seen in the supernatants and the membrane fractions but not in the cytoplasm at three days [27]. It was possible to see only very small amounts of TcdB1 using the TcdB1^N-terminus^ antibody in the membrane fraction only however. We hypothesized that this reflects a tight regulation between translation and export, and that the TcdB1 N-terminus is involved in this process. In support of this hypothesis, removal of the N-terminus of the B-subunit strongly reduced the production level of not only the B but also of the C-subunit. Taken together these observations suggest that under native expression level conditions, translation of the N-terminal domain of the B-subunit is rate limiting to synthesis and secretion and that this domain may also be removed during secretion.

A previous report suggested that a B-subunit from *Yersina* (TcaC) could be secreted via a type III secretion system [34]. We argue this is not the normal route of export for *Photorhabdus* homologues, as our fully functional *E. coli* secretion model contains no type III system genes. Nevertheless a comparison of the amino acid sequences of TcaC from *Yersinia pestis* and TcdB1 from *Photorhabdus* show good identity along the length of the protein. As *Photorhabdus* encodes a type III secretion system it remains possible that B-subunits could also be secreted via this system.

Our evidence suggests that the N-terminus of the C-subunit is responsible for export of the complex across the inner membrane. In this model, the C-subunit fulfils the analogous role of the type II secretion leader peptide of SpvB. We can draw further analogy between SpvB and the B+C sub-complex. The SpvB protein has a Type III secretion-independent N-terminal domain, fused by a proline stretch to a “toxic” ADP-ribosyltransferase C-terminal function. In the case of the B+C sub-complex, the N-terminal domains of the B and C-subunits provide the export system. The OspA-like domains of the B and C–subunits likely form the protein-protein interaction domains of the sub-complex while the variable C-termini of the C-subunit constitutes the active “toxin” domain. In the case of TccC5 this also constitutes an ADP-ribosyltransferase domain involved in forcing actin clustering [14]. Figure 9B shows a diagrammatic summary of the roles that the different subunit domains play in the export process.

It was surprising that the C-subunit is able to export the A-subunit in the absence of the B-subunit. It is possible that this relies on the injection-like mechanism of TcdA1 as recently proposed by Gatsogiannis et al [13]. The A-subunit does not possess an OspA-like structural domain although previous studies have identified protein-protein interaction domains in the C-terminus [3] [13]. While there is no evidence to support this, we might speculate that the SpvA-like N-terminus of TcdA1 is able to substitute for the SpvB-like N-terminus of TcdB in this case.

It was interesting that the over-production of truncated TccC5 proteins lacking the toxin C-terminal domain in *P. luminescens* W14 was able to supress oral toxicity of the supernatant. Different hypotheses may be proposed to explain this. Firstly, the over-expressed truncated subunits might be interfering with the normal export pathways, essentially blocking the export of the native orally toxic Tca and Tcd complexes. Alternatively the truncated subunits may be replacing the native full-length C-subunit proteins, producing predominantly non-toxic TC derivatives. If the latter were the case then it would suggest that the C-subunits are relatively promiscuous regarding which Toxin Complexes they interact with, in this case both Tca and Tcd. The initial work on Tca and Tcd in strain W14 suggested that they form distinct complexes, with Tca comprising TcaA, TcaB and TcaC and Tcd comprising TcdA1 and TcdB1. Interestingly in these earlier studies the C-subunit proteins were not detected [5], [35], [36].

In conclusion we have investigated the role of various domains of TC subunits in secretion. Central to secretion are the N-terminal domains of the two subunits of the B+C sub-complex. These domains serve to export the TC in the absence of any other specialised *Photorhabdus* proteins. This suggests either that they encode a “self-contained” export system or that they interact with an as yet undefined export system also present in laboratory strains of *E. coli*. Furthermore, the presence of B and C-subunit gene homologues in a range of diverse bacterial species indicates that this toxin secretion mechanism is not restricted to the insect pathogens.

## Supporting Information

Figure S1 **The cosmid model for heterologous TC synthesis and secretion in** ***E. coli*** **.** The data illustrates oral bioassays of cell free supernatants from the cosmid model including the various transposon insertion mutant clones. The mean larval weight (MLW) gain of cohorts of *M. sexta* neonates (n = 30) is shown. Supernatants are from 2 day old cultures grown at 28°C in LB medium. Note the A1, B1 and C5-subunits are all required for supernatant toxicity.
(TIFF)

Figure S2 **The C-terminal domain of the C-subunit is required for toxicity.** The A-subunit and B+C sub-complex complementation bioassays show that the C-terminal tail of the C-subunit is necessary for oral toxicity. Mean larval weight (MLW) gain of cohorts of *M. sexta* neonates (n = 10) fed dilutions of mixed sonicated cell extracts (L) from bespoke heterologous production strains. Samples were mixed at a ratio of 1∶1 (50 µl each) and diluted 1∶40 in PBS buffer. The more potent the toxic effect, the smaller the mean larval weight. Error bars represent the standard error. A key using the data column fill pattern is given above the graph to assist in the interpretation of the predicted TC subunit contents of the test samples.
(TIFF)

Figure S3 **Overexpression of C-terminal truncated copies of** ***tccC5*** **interferes with normal oral toxin production in** ***P. luminescens*** **W14.** Inducible expression of C-terminally truncated copies of the C-subunit (Fig. 5A) in the original *P. luminescens* W14 strain leads to a significant reduction in oral toxicity of the culture supernatant to *M. sexta* neonates. Supernatants were taken 2 days post induction. Mean larval weight (MLW) gain of cohorts of *M. sexta* neonates (n = 10) fed 1∶5 dilutions of culture supernatants. Error bars represent the standard error. Note the black filled columns indicate normal levels of supernatant toxicity. White filled columns show a reduction of toxicity associated with induction of the truncated *tccC5* gene.
(TIFF)

Figure S4 **Full length TccC5 protein shows toxicity to** ***Galleria*** **.** (**A**) *tccC5* expression constructs in pBAD30 with N- and C-terminal His-tags (pC5_N_his and pC5_C_his) and a C-terminal truncation (pC5Sm_his). (**B**) Percentage mortality of *Galleria* larvae after injection (n = 8). Note full-length protein showed some toxicity (black filled) while the C-terminally truncated construct showed none (hatched).
(TIFF)

Figure S5 **Co-production of B-subunit (TcdB1) and C-subunit (TccC1) proteins confirms they tightly associate in the** ***E. coli*** **cytoplasm.** (**A**) Construction of two expression plasmids designated pBC1 and pB. (**B**) 2D gel analysis of total cell protein demonstrates that the B and C bind together (arrows) as confirmed MALDI-TOF analysis of these protein spots.
(TIFF)

Text S1 **Two dimensional electrophoresis method.** The method used to visualize the tight binding of the TcdB1 and TccC1 subunit proteins illustrated in figure S1.
(DOCX)
