# Supplementary material for: The Role of TcdB and TccC Subunits in Secretion of the Photorhabdus Tcd Toxin Complex
Source: PLoS Pathog. 2013 Oct 3;9(10):e1003644. doi: 10.1371/journal.ppat.1003644 (PMC3789776; doi:10.1371/journal.ppat.1003644)
Supplement: Text S1 — Two dimensional electrophoresis method. The method used to visualize the tight binding of the TcdB1 and TccC1 subunit proteins illustrated in figure S1. (DOCX) [file ppat.1003644.s006.docx]

**Text S1: Two-dimensional gel electrophoresis***: E. coli* TC-subunit heterologous production strains containing either pB1 or pB1C1 were induced overnight with 0.2% (w/v) L-arabinose. Total cellular protein was extracted using phenol precipitation and re-suspended in 150μl CDU buffer (4% CHAPS, 130mM DTT and 9M Urea) containing 1 x HALTTM protease Inhibitor Cocktail Mix (Pierce, Thermo Fisher, UK). Samples were incubated for 2 h at room temperature, before centrifugation for 30 min at 88 760X g. A protein quantitation kit (Molecular Probes, Invitrogen) was used to quantify protein concentration in the samples and equivalent amounts of total proteins were loaded. A Multiphor isoelectric focusing and horizontal SDS-polyacrylamide gel electrophoresis system, using Immobiline DryStrip gels and precast 12.5% SDS gels were used following the GE Healthcare manufacturer’s instructions. Gels were Coomassie-blue stained and protein spots were excised and sent to the protein sequencing facility at the University of the West of England (Bristol, UK). The peptide sequences resulting from MALDI analysis of trypsin-digested protein, were compared to TC proteins using MASCOT.
